# Evaluating the pharmacodynamic effects of padsevonil in healthy volunteers using simultaneous [^11^C]-UCB-J PET and MR Arterial Spin Labeling measurements

**DOI:** 10.1016/j.nsa.2023.101133

**Published:** 2023-08-19

**Authors:** Michel Koole, Brigitte Lacroix, Chunmeng Tang, Hugues Chanteux, Ralph Paul Maguire, Koen Van Laere

**Affiliations:** a Nuclear Medicine and Molecular Imaging, Department of Imaging and Pathology, KU Leuven, Belgium; b UCB Pharma, Braine l’Alleud, Belgium; c Division of Nuclear Medicine, University Hospitals Leuven, Leuven, Belgium

**Keywords:** Simultaneous PET-MR, Padsevonil, Drug occupancy, rCBF changes, Pharmacodynamic effects

## Abstract

**Introduction:**

Integrated PET-MR scanners can measure simultaneously tracer binding, downstream cerebral blood flow (CBF) and neuronal activation. An integrated GE Signa PET-MR system was used to combine [^11^C]-UCB-J PET scanning with MR Arterial Spin Labeling (ASL) measurements to evaluate the effects of padsevonil (PSL) on SV2A occupancy and CBF simultaneously.

**Methods:**

PET-MR scanning was performed in 10 healthy controls (8M/2F; 27.6 ​± ​10.0 ​yrs) at baseline, and at two timepoints (post1: 2−24h and post2: 6−30h) after administration of a single PSL dose (6.2−100 ​mg). Dynamic [^11^C]-UCB-J PET scanning with arterial blood sampling was performed to estimate distribution volumes and corresponding SV2A occupancies. Simultaneously, MR ASL data were acquired and spatially normalized to Montreal Neurological Institute (MNI) space for voxel wise analysis while brain Volume-Of-Interest (VOI) were defined using a simplified Hammers atlas.

**Results:**

PET measurements showed a SV2A occupancy range of 57–98% and 16−63% and a corresponding PSL plasma concentration range of 3.6–189.23 ​ng/mL and 1.3–9.8 ​ng/mL for post1 and post2, respectively. When comparing post1 to baseline ASL measurements, a VOI-based analysis detected a significant CBF decrease in thalamus, insula, cerebellum, posterior cingulate cortex, and brain stem, which was confirmed by a voxel-wise analysis comparing baseline and post1 ASL measurements and identifying significantly different clusters covering the same brain regions. In addition, we demonstrated CBF recovery in these brain regions for the post2 ASL measurements compared to baseline measurements. For the thalamus, a VOI-based correlation analysis detected a significant correlation between PSL plasma concentration and CBF decrease relative to baseline conditions.

**Discussion:**

A pattern of local and dose dependent CBF decrease was demonstrated following PSL administration. More generally, these findings present the potential of simultaneous PET and ASL measurements to follow the time course of drug effects and to differentiate between the efficacy of different drugs in patient groups.

## Highlights

• Simultaneous PET-MR ASL measurements showed a pattern of locally decreased CBF after a single dose of padsevonil.
• Regional CBF changes proved to be dose dependent.
• Pharmacological PET-MR can demonstrate target engagement of novel CNS agents and provide evidence of efficacy and safety at an early stage.

## Graphical abstract

**Figure:**
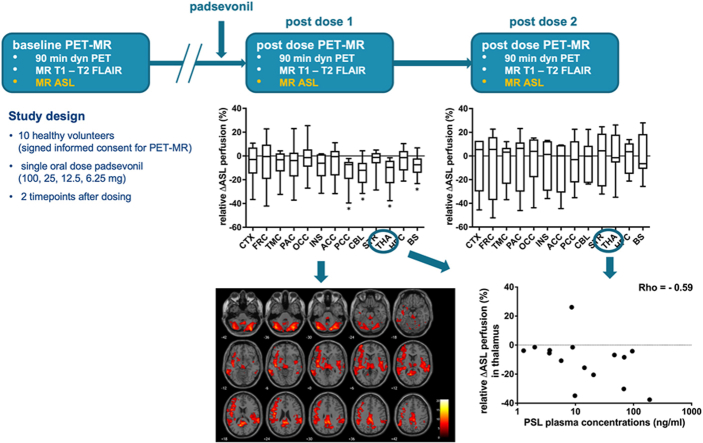

## 1 Introduction

Novel pharmaceutical medicines remain the key therapeutic approach for addressing the growing challenges posed by brain disorders. To accelerate the development of new CNS medicines quantitative brain Positron Emission Tomography (PET) is an essential tool to demonstrate receptor occupancy and target engagement of new therapeutics at different dose levels and optimize human dosing regimens for proof of clinical concept trials (Matthews et al., 2012). As such PET plays an important role in early go/no-go decision-making and de-risking strategies to reduce drug attrition rates. However, quantitative PET imaging generally requires dynamic scanning procedures with arterial blood sampling which can be logistically challenging and increase costs significantly. It also implies the availability and administration of a selective PET radiolabeled molecule and comes with limited additional radiation exposure for patients or healthy volunteers.

In addition to PET imaging, pharmacological magnetic resonance imaging (phMRI) (Lanfermann et al., 2015), (Honey and Bullmore, 2004) has been used to investigate regional, hemodynamic changes associated with specific brain activity in response to CNS-active drugs. Contrary to PET, phMRI uses endogenous contrast enhancement methods to provide accessible and cost-effective pharmacodynamic assays which could be used to establish brain-penetrability parameters, or dose-ranging information for novel therapeutic compounds (Leslie, 2000).

With the introduction of integrated PET-MR systems, PET and MR measurements can be performed simultaneously to gain insight into both the pharmacokinetic and pharmacodynamic properties of a new drug (Sander et al., 2020). However, limited imaging studies have taken advantage of the simultaneous PET-MR measurements for the pharmacological evaluation of new CNS drugs with phMRI mainly using functional MRI (fMRI) and the BOLD paradigm to evaluate drug-induced changes in brain function.

For this study, dynamic PET-MRI scanning was performed simultaneously to evaluate the effect of a single dose of Padsevonil (PSL, UCB Pharma, Brussels, Belgium) on the occupancy of Synaptic vesicle glycoprotein 2A (SV2A) using [^11^C]-UCB-J as PET and on cerebral blood flow (CBF) using MR Arterial Spin Labeling (ASL) CBF measurements (Ferré et al., 2013), (Detre et al., 2009). PSL is an investigational new drug for epilepsy with a selective affinity for all 3 subtypes of the human presynaptic synaptic vesicle 2 (hSV2) protein (ie, SV2A, SV2B, and SV2C) and the postsynaptic central benzodiazepine receptor (cBZR) sites on the gamma-aminobutyric acid (GABAA) receptor (Wood et al., 2020). PET based SV2A occupancy estimates have already been reported using [^11^C]-UCB-J as PET radioligand which has shown high affinity and specificity for SV2A, and optimal pharmacokinetic properties with no evidence of brain penetrating radiometabolites (Finnema, 2016). Next to the dose dependent SV2A occupancies which were already estimated for PSL with [^11^C]-UCB-J PET (Muglia, 2020), the aim of this study was to identify brain areas with PSL-induces changes by comparing ASL CBF measurements prior to dosing with measurements after drug intake. In addition, we wanted to determine whether changes in neuronal activity in neuronal activity following PSL administration were dose dependent.

## 2 Experimental procedures

### 2.1 Study design

In total, 10 carefully screened healthy volunteers (8 males and 2 females, age 27.6 ​± ​10.0 years (mean ​± ​SD) with an age range of 20–54 years) underwent baseline PET-MRI scanning. All subjects received a single oral dose of padsevonil ranging from 6.25 up to 100 ​mg. Post drug PET-MRI scanning was performed at 2h (7 scans), 6h (4 scans), 24h (4 scans), 27h (1 scan) and 30h (2 scans) after drug administration (see Table 1). Dose range and timing of the post drug PET-MRI scans were based on the adaptive design of this study to achieve different plasma levels of the drug and therefore different exposure and occupancy levels in the brain (Takano, 2016). As such, overall occupancy estimates of 65.3 ​± ​26.8% (mean ​± ​SD) were obtained with a 16%–98% range. For the first post drug scans (n ​= ​10), occupancy estimates were 82.1 ​± ​14.5% with a range of 57%–98%, while for the second post drug scans (n ​= ​7), occupancy values were 41.2 ​± ​21.2% with a 16%–63% range. This way, an optimal sampling of the dose occupancy curve was achieved. In total, 17 post dose PET-MRI scans were performed with 2 post drug PET scans performed for 7 subjects and only 1 post dose PET scan performed for 3 subjects because of scanner or tracer failure or because of problems with the arterial blood sampling procedure.

**Table 1:** Overview of the dosing and scanning scheme of the adaptive PET/MR dose occupancy study.

	PSL Dose	Post dose 1		Post dose 2	
Subject		Time post dose	Occupancy	Time post dose	Occupancy
01	100 ​mg	24 ​h	69.6%	–	–
02	25 ​mg	2 ​h	86.0%	–	–
03	100 ​mg	6 ​h	96.7%	30 ​h	61.5%
04	100 ​mg	6 ​h	95.8%	30 ​h	66.2%
05	100 ​mg	2 ​h	99.3%	24 ​h	62.2%
06	25 ​mg	2 ​h	87.4%	27 ​h	2.2%
07	12.5 ​mg	2 ​h	91.6%	–	–
08	12.5 ​mg	2 ​h	73.0%	24 ​h	12.5%
09	6.25 ​mg	2 ​h	56.4%	6 ​h	23.3%
10	6.25 ​mg	2 ​h	66.5%	6 ​h	46.6%

The study was approved by the Ethics Committee for Research of the University Hospital Leuven where the study took place. Informed consent was obtained from all individual participants included in the study.

### 2.2 [^11^C]UCB-J PET/MR imaging

PET-MRI scans were acquired with the integrated GE Signa PET/MR system with the PET tracer synthesis, data acquisition and reconstruction parameters described in an earlier report (Koole, 2019). Data acquisition and processing were performed with GE Signa PET/MR MP24 software release.

Simultaneous with the 90 ​min dynamic PET scan, both a 3D volumetric T1-weighted BRAVO sequence (plane: sagittal; TE: 3.2ms; TR: 8.5ms; TI: 450ms; Flip Angle: 12; Receiver Bandwidth: 31.2; NEX: 1; voxel size: 1x1x1 mm) and 3D T2-weigthed CUBE FLAIR sequence (plane: sagittal direction; TE: 137ms; echoes: 1; echo train length: 190; TR: 8500ms; TI: 50ms; Receiver Bandwidth: 31.25; NEX: 1; voxel size: 1.2x1.3 ​× ​1.4 ​mm) were acquired.

In addition, a GE proprietary Enhanced Arterial Spin Labeling (eASL) sequence was acquired. This is a 3D pseudo continuous ASL sequence (PCASL) with 3 different post label delay times encoded into a single acquisition (Dai et al., 2008), (Dai et al., 2013). Images with post label delay times of [1.00, 1.57, 2.46] seconds and effective label durations of [0.57, 0.89, 2.04] seconds were reconstructed to span a broad range of short, medium, and long transit delays. For this sequence, the MR image field of view (FOV) was placed from the top of the skull to the lower portion of the cerebellum while the labeling plane was approximately 2 ​cm below this imaging slab. Scan parameters included an axial FOV of 22 ​× ​22 ​cm, an acquisition matrix of 6 arms with 800 sampling points, a 62.5-kHz bandwidth, 26 slices with a 5.5 ​mm slice thickness, an echo time of 12.4 ms, a repetition time of 7.910 ​s, and a NEX (no. of excitations) of 3. This resulted in an acquisition time of 12 ​min. Each slice was regridded into a 128 ​× ​128 matrix with a pixel size of 1.72 x 1.72 ​mm. Transit time–corrected CBF maps were calculated by incorporating the combined delay image and the transit delay with the combined delay map consisting of the sum of the delay times per subject while a transit-delay map reflecting the transit time of blood from the labeling plane to the imaging plane was estimated using a signal-weighted delay method (van der Thiel, 2018).

PSL plasma concentrations were recorded in ng/mL at the start, the middle, and the end of each post dose PET-MRI scan.

### 2.3 Data processing

Post dose ASL data were rigidly co-registered (only translation and rotation parameters) with baseline ASL data, after which baseline and post dose ASL datasets were used to generate an average ASL dataset. This average ASL dataset was then used to estimate the optimal rigid transformation to align all ASL data with the baseline anatomical high-resolution 3D T1 BRAVO. Finally, the latter was used to estimate the optimal non-linear transformation to spatially normalize all ASL data to Montreal Neurological Institute (MNI) space using SPM8 (default settings). Brain Volume of Interests (VOIs) were defined using a simplified Hammers atlas and restricted to gray matter by applying a simple threshold of 0.3 to the individual gray matter probability maps. For this purpose, a Statistical Parametric Mapping based multichannel segmentation (SPM12, standard settings) using both the 3D T1 BRAVO and interpolated T2 CUBE FLAIR was applied to determine subject specific tissue probability maps for gray matter. This way, brain VOIs for the global cortex (CTX), frontal (FRC), temporal (TMC), parietal (PAC) and occipital cortex (OCC) as well as the insula (INS), anterior (ACC) and posterior cingulate (PCC), striatum (STR), thalamus (THA), hippocampus (HPC), cerebellum (CBL), and brainstem (BS) were determined.

This set of brain VOIs was used to extract regional CBF estimates from both baseline and post dose ASL data which were smoothed with an isotropic Gaussian filter kernel (8 ​mm full width half maximum – FWHM) prior to the VOI-based analysis to reduce the effect of noise. For the voxelwise analysis, post dose ASL data were additionally masked using a binary brain mask and normalized to the baseline ASL data.

The average padsevonil plasma concentration for a post dose PET-MR scan (C_avg_) was calculated from the samples at the start, the middle, and the end of the post-dose PET-MR scan as C_avg_ ​= ​AUC/Δt with AUC ​= ​area under the concentration vs time curve calculated using the trapezoidal rule and Δt ​= ​difference between the time at the end and at the start of the post dose PET-MRI scan.

For C_avg_, the lower limit of quantification was set to 0.5 ​ng/mL. Based on the PET based SV2a RO and corresponding PSL C_avg_, a population pharmacokinetic-pharmacodynamic (PKPD) analysis was performed (NONMEM 7.3.0, ICON Dev. Solutions) to estimate the population EC_50_ and EC_90._

### 2.4 Statistical analysis

A VOI based analysis was performed to evaluate regional changes in CBF relative to baseline. To evaluate whether relative differences were significantly different from zero, a Wilcoxon signed rank test (p ​< ​0.05) was used. In addition, we determined the effect size as Cohen's d representing the difference between the averages of baseline and each of the blocking conditions divided by the pooled standard deviation. To evaluate a dose dependent effect, a Spearman's rho correlation (p ​< ​0.05) was performed between regional CBF differences relative to baseline and PSL C_avg_. In addition to the VOI-based analysis, a voxelwise analysis was performed using SPM12 (Statistical Parametric Mapping, University College London, UK) to detect clusters of voxels (p_uncorr_ < 0.001, k_ext_ > 500 voxels) with statistically significant CBF differences between baseline and blocking conditions using a paired *t*-test and with a statistically significant linear relation between relative CBF differences and PSL plasma concentrations using a regression analysis.

A post-hoc analysis of the correlation between CBF change and PK concentration was performed for the thalamus region of interest, across subjects and scan timepoints using a non-parametric (spearman) test.

## 3 Results

Average PSL C_avg_ was 36.46 ​± ​51.76 ​ng/ml with a range of 1.28–189.23 ​ng/mL. Two plasma concentrations corresponding to the lower PET-based RO estimates of 16 and 18% were below the limit of quantification 0.5 ​ng/mL and excluded for further analysis, leaving a total of N ​= ​15 ​PK data points For the first post drug scans (n ​= ​10), average PSL C_avg_ was 56.4 ​± ​59.6 ​ng/mL with a range of 3.6–189.23 ​ng/mL while for the second post drug scans (n ​= ​5), average PSL C_avg_ was 5.1 ​± ​3.9 ​ng/mL with a range of 1.3–9.8 ​ng/mL.

Using the E_max_ model, the population EC_50_ for PSL was estimated as 3.1 ​ng/mL with a 95% confidence interval of [2.0–4.2], while the estimated EC_90_ was 27.9 ​ng/mL with a 95% confidence interval of [18.1–37.7].

**Fig. 1:**
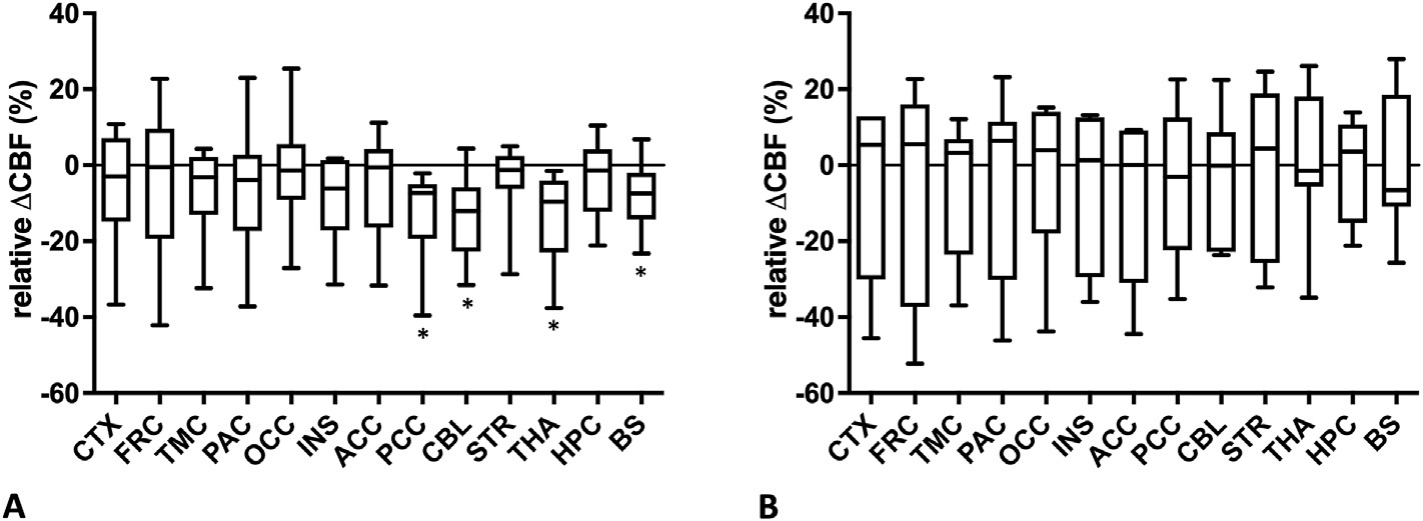
Box Whisker plots of regional CBF differences relative to baseline as measured with ASL MRI after oral administration of a single PSL dose for the first post dose scans (N ​= ​10) with 82% avg SV2A RO (A) and second post dose scans (N ​= ​7) with 41% avg SV2A RO (B). A statistically significant decrease (Wilcoxon signed rank test, p ​< ​0.05, marked with ∗) of CBF relative to baseline was observed in PCC, CBL, THA, and BS for the first post dose scans while no statistically significant increase was detected in other brain regions. For the second post dose scan, neither a statistically significant increase nor decrease of CBF relative to baseline was observed in any brain region. (CTX ​= ​cortex, FRC ​= ​frontal cortex, TMC ​= ​temporal cortex, PAC ​= ​parietal cortex, OCC ​= ​occipital cortex, INS ​= ​insula, ACC ​= ​anterior cingulate cortex, PCC ​= ​posterior cingulate cortex, CBL ​= ​cerebellum, STR ​= ​striatum, THA ​= ​thalamus, HPC ​= ​hippocampus, BS ​= ​brainstem).

Based on the VOI-based analysis, a statistically significant decrease of brain CBF relative to baseline was observed in the PCC (p ​= ​0.0020), CBL (p ​= ​0.0059), THA (p ​= ​0.0020), and BS (p ​= ​0.0195) for the first post dose scans (N ​= ​10, 82% avg SV2A RO) while no statistically significant increase was detected (see Fig. 1A). For the second post dose scans (N ​= ​7, 41% avg SV2A RO), neither a statistically significant CBF increase nor decrease relative to baseline was observed (see Fig. 1B). These findings were confirmed by the Cohen's d effect sizes (see Table 2) showing a much higher effect on CBF for the first blocking conditions compared to the second blocking conditions. In addition, brain regions demonstrating statistically significant differences in CBF between baseline and blocking conditions, corresponded to the highest effect sizes.

**Table 2:** Effect sizes (Cohen's d) for the differences in ASL CBF measurements between baseline and first post dose scan and between baseline and second post dose scan. (CTX ​= ​cortex, FRC ​= ​frontal cortex, TMC ​= ​temporal cortex, PAC ​= ​parietal cortex, OCC ​= ​occipital cortex, INS ​= ​insula, ACC ​= ​anterior cingulate cortex, PCC ​= ​posterior cingulate cortex, CBL ​= ​cerebellum, STR ​= ​striatum, THA ​= ​thalamus, HPC ​= ​hippocampus, BS ​= ​brainstem).

post dose	CTX	FRC	TMC	PAC	OCC	INS	ACC	PCC	CBL	STR	THA	HPC	BS
first	0.41	0.40	0.44	0.47	0.16	0.70	0.43	0.71	0.67	0.34	0.75	0.38	0.47
second	0.13	0.17	0.17	0.06	0.03	0.28	0.34	0.13	0.03	0.06	0.01	0.00	0.02

**Fig. 2:**
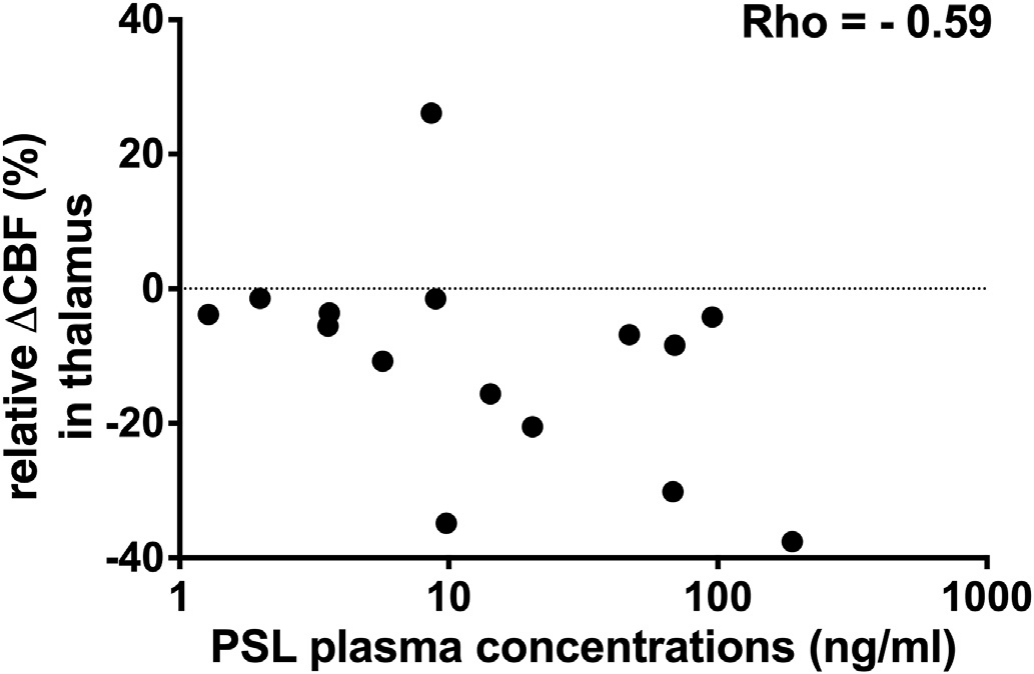
Scatter plot of the ASL CBF differences relative to baseline for the (bilateral) thalamus as function of the PSL plasma concentrations (N ​= ​15).

In terms of a dose dependent effect, only a significant correlation (p ​= ​0.0242) was found for THA between CBF differences relative to baseline and the PSL plasma concentrations (Rho ​= ​−0.59, 95% confidence interval ​= ​[−0.85, −0.09]) (see Fig. 2).

**Fig. 3:**
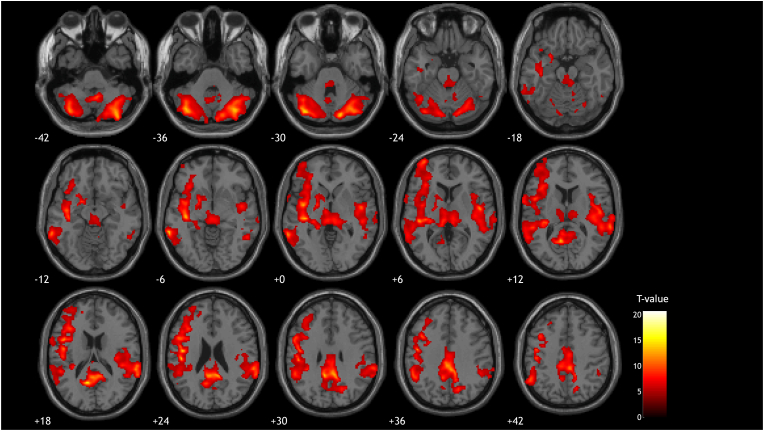
SPM T-maps showing the statistically significant clusters (paired T-test, p_uncorr_ < 0.001, k_ext_ > 500 voxels) when comparing baseline and first post drug MR ASL scans (N ​= ​10) after a single oral PSL dose. The average PSL plasma concentration for the post drug scan was 56.4 ​± ​59.6 ​ng/mL with a range of 3.6–189.23 ​ng/mL.

In terms of a voxel wise analysis, statistically significant clusters were found (N ​= ​10, paired T-test, p_uncorr_ < 0.001, k_ext_ > 500 voxels) when comparing baseline and first post dose scans (see Fig. 3). These clusters include CBL, THA, PCC and BS which is in line the VOI-based analysis. On the other hand, no significant clusters were detected between the baseline and second post dose. In addition, a regression analysis didn't reveal any clusters demonstrating a signification linear relation with PSL C_avg_.

## 4 Discussion

This is the first study to combine synaptic density PET imaging and MRI ASL to simultaneously evaluate SV2A occupancy and downstream pharmacodynamic effects for the same PSL exposure. The potential of simultaneous PET-MR imaging for neuropharmacology has already been demonstrated by previous PET-MR studies (Sander et al., 2020), (Bruinsma et al., 2018)– (Sander et al., 2013). In addition, simultaneous PET-fMRI was used to characterize dynamic neuroreceptor adaptations in vivo, such that PET-fMRI could be considered as a non-invasive method for assessing D2/D3 receptor desensitization and internalization (Sander et al., 2016). Next to the dopamine system, simultaneous PET-MRI was used to investigate the effects of functionally different compounds on the 5-HT_1B_ receptor occupancy using [^11^C]AZ10419369 and the associated hemodynamic responses with fMRI in anaesthetized male nonhuman primate (Hansen et al., 2017). In addition, Vidal et al. used simultaneous PET-MRI for the exploration of 5-HT_1A_ receptor occupancy using [^18^F]MPPF PET and its consequences in terms of brain activation using fMRI in anaesthetized cats, and demonstrated differential signaling by two 5-HT_1A_-biased agonists (Vidal et al., 2018). On the other hand, simultaneous PET and pharmacological fMRI has been used to evaluate the acute pharmacological response to selective serotonin reuptake inhibitors in the human brain (Gryglewski et al., 2019). However, while an average 5-HTT occupancy of 69% was observed with PET, fMRI was not able to detect significant differences in the pharmacological response between selective serotonin reuptake inhibitors (SSRI) and placebo scans, although SSRI effects were reported in previous fMRI studies (McKie, 2005). As a result, the authors questioned whether the analysis pipeline to explore acute SSRI effects with pharmacological resting state fMRI was appropriate or whether the study design should be reconsidered since it was shown that test–retest reliability of CBF assessed with ASL was higher compared to BOLD in certain applications (Holiga et al., 2018).

For this study, we used a hybrid PET-MR system to simultaneously measure SV2A occupancies using synaptic density PET imaging and relative CBF changes using MR ASL for different plasma levels of PSL. Similar to regional CBF measurements with [^15^O]H_2_O PET, ASL is based on the flow of spin-labelled water into brain tissue, rather than changes in magnetization of the blood in vessels immediately adjacent to the tissue – as with fMRI.

Based on the MR ASL measurements, we observed a reduced CBF in the posterior cingulate cortex, thalamus, brainstem, and cerebellum after a single oral dose of PSL. This was confirmed by both a VOI-based and voxel wise comparison of baseline and first post dose MR ASL measurements where the first post dose scans corresponded to an average SV2A occupancy of around 82% and average PSL plasma concentration of around 56 ​ng/ml. The observed pattern of decreased regional CBF was very similar to the pattern of reduced regional CBF observed during human benzodiazepine-induced non-rapid-eye-movement (non-REM) sleep which was determined with [^15^O]H_2_O PET imaging (Hiroki et al., 2006). This latter pattern consisted of a reduced CBF bilaterally in the frontal, parietal, and temporal neocortical regions; orbital basal forebrain; cingulate gyrus; insular cortex; thalamus; and cerebellar hemisphere. This similarity between both patterns could indicate that the effects observed by ASL after a single oral dose of PSL are due to the transient GABAA receptor occupancy. Indeed, PSL was synthetized to ensure differential proportionality of target engagement with quasi full saturation of SV2A and low GABA_A_R occupancy (Wood et al., 2020). However, while > 90% SV2A occupancies were reached, [^11^C]-Flumazenil PET studies (Abadie, 1992) still showed a 10–15% transient GABA_A_R occupancy where PSL acts as a partial agonist at the benzodiazepine site. On the other hand, the GABA_A_R occupancy in healthy controls was generally reduced to around 5% at 2 ​h post dose for PSL doses of 200 and 400 ​mg (Muglia et al.) which were much higher than the PSL doses in this study, such that for this study even lower GABA_A_R occupancy levels are to be expected. However, even a small GABA_A_R engagement could still contribute to this pattern of decreased regional CBF comparable to a pattern of reduced wakefulness (Grimwood and Hartig, 2009). In addition, an effect from SV2B and/or SV2C occupancy by PSL can't be excluded.

Since the observed effects are like those that have been observed in sedative or somnolence induced by GABA_A_R treatments, it cannot be ruled out that they are due to sedative effects alone. Potentially using an independent measure of sedative effects, e.g. EEG might help to confirm whether the effects are due to underlying changes in brain state. Future studies could examine whether somnolence, induced by other pharmacological mechanisms or indeed natural somnolence or sleep induces similar regional CBF patterns of activation/deactivation in the brain.

No statistically significant differences in CBF were observed between the baseline and second post dose scan which corresponded to a much lower average SV2A occupancy of around 41% and a much lower average PSL plasma concentration of around 5 ​ng/ml. This can be explained because of the decreased concentration at the second post-dose scan, relative to the first post-dose scan and confirms a transient pharmacological effect on CBF which is correlated with the transient PSL pharmacokinetics.

This relationship was supported by the VOI-based analysis where a significant negative correlation was found between 10.13039/501100009517PSL plasma concentrations and change in CBF in the thalamus. This correlation was performed across all the PK and ASL data points available, so that data from some subjects contributed 2 data points to the analysis. Since the second timepoint did not show a significant change, this meant that many of the data points were close to zero ASL change. With only two data points per subject, we did not model the potential for a within-subject effects in the magnitude of change in the thalamus region and this could potentially be addressed by a larger dataset.

A voxel wise analysis did not show any significant clusters of voxels with a linear relationship between the PSL plasma concentration and decrease of regional CBF, possibly because the number of datapoints is too small for a mass-univariate analysis with good statistical power. Although it was not the focus of this study, we also performed a VOI-based correlation analysis between PET-based SV2A occupancies and corresponding regional CBF decreases measured with MR ASL, which confirmed the thalamus and posterior cingulate cortex as brain regions demonstrating a significant Spearman correlation. In addition, a voxel wise regression analysis identified statistically significant clusters of voxels demonstrating a linear relationship between SV2A occupancy and regional CBF decrease. The identification of significant clusters of regional CBF decrease using occupancy instead of PSL plasma concentration could be explained by the nonlinear relation of occupancy vs. PSL plasma concentration. However, these findings further supported a concentration dependent effect of 10.13039/501100009517PSL on regional CBF.

In a broader context, the simultaneous measurements of regional CBF with MR ASL and receptor occupancy with PET, allows to anticipate the potential impact of drug-induced changes in regional CBF on the quantification of the PET tracer uptake, especially for PET tracers demonstrating irreversible tracer kinetics. For these PET tracers, brain uptake is not only driven by the density of available binding targets but also partially dependent on blood flow such that quantification can be susceptible to delivery effects (Koeppe et al., 1994), (Koeppe et al., 1999). Also, where using simplified standard uptake value (SUV) quantitation methods it is important to understand if there has been a change in regional CBF.

Finally, although this was a rather extensive study in terms of a PET dose occupancy study with 10 baseline and 17 post dose PET scans in total, the number of MR ASL datasets is rather limited from a MRI perspective, which could be considered as a limitation of this study. Moreover, the adaptive design of this study with multiple PSL doses to determine the PSL plasma concentration vs SV2A occupancy relationship and guide dose selection for future clinical trials, resulted in a wide range of PSL plasma concentrations. Future studies could incorporate multiple ASL measurements at each scan timepoint or longer ASL sequences to improve the precision of the regional CBF estimates. In addition, the sequence of scanning under baseline and blocking conditions was not randomized such that a potential bias because of the first scanning session being consistently the scanning session under baseline conditions could not be evaluated. Nevertheless, we were able to determine a significant effect of PSL on regional CBF and in addition, we could demonstrate that this effect was concentration dependent.

## Contributors

Brigitte Lacroix, Hugues Chanteux, Ralph Paul Maguire and Koen Van Laere designed the study and wrote the protocol. Michel Koole, Chunmeng Tang, Brigitte Lacroix and Ralph Paul Maguire performed the data analysis. Michel Koole and Ralph Paul Maguire wrote and finalized the manuscript. All authors contributed to and have approved the final manuscript.

## Role of funding source

Part of this study was funded by a UCB Pharma research grant to KU Leuven (principal investigator Koen Van Laere). UCB Pharma helped with the design of the study and interpretation of the data. In addition, UCB Pharma supported the writing of the paper and approved the submission for publication. UCB Pharma had no role in the collection and analysis of the data.

## Declaration of competing interest

Brigitte Lacroix, Hugues Chanteux and Ralph Paul Maguire are employees of UCB Pharma. All other authors declare that they have no conflicts of interest.
